# Preoperative vs Postoperative Opioid Prescriptions and Prolonged Opioid Refills Among US Youths

**DOI:** 10.1001/jamanetworkopen.2024.20370

**Published:** 2024-07-05

**Authors:** Tori N. Sutherland, Jennifer A. Rabbitts, Gregory E. Tasian, Mark D. Neuman, Craig Newcomb, Scott E. Hadland

**Affiliations:** 1 Department of Anesthesiology and Critical Care, Children’s Hospital of Philadelphia (CHOP), University of Pennsylvania Perelman School of Medicine, Philadelphia; 2 Leonard Davis Institute of Health Economics, University of Pennsylvania, Philadelphia; 3 Center for Perioperative Outcomes Research and Transformation, University of Pennsylvania Perelman School of Medicine, Philadelphia; 4 Department of Anesthesiology, Perioperative, and Pain Medicine, Stanford University School of Medicine, Palo Alto, California; 5 Division of Urology, Department of Surgery, Children’s Hospital of Philadelphia, Philadelphia, Pennsylvania; 6 Department of Biostatistics, Epidemiology, and Informatics, University of Pennsylvania, Philadelphia; 7 Department of Anesthesiology and Critical Care, University of Pennsylvania Perelman School of Medicine, Philadelphia; 8 Center for Clinical Epidemiology and Biostatistics, University of Pennsylvania Perelman School of Medicine, Philadelphia; 9 Division of Adolescent and Young Adult Medicine, Mass General for Children, Boston; 10 Department of Pediatrics, Harvard Medical School, Boston, Massachusetts

## Abstract

**Importance:**

High-risk practices, including dispensing an opioid prescription before surgery when not recommended, remain poorly characterized among US youths and may contribute to new persistent opioid use.

**Objective:**

To characterize changes in preoperative, postoperative, and refill opioid prescriptions up to 180 days after surgery.

**Design, Setting, and Participants:**

This retrospective cohort study was performed using national claims data to determine opioid prescribing practices among a cohort of opioid-naive youths aged 11 to 20 years undergoing 22 inpatient and outpatient surgical procedures between 2015 and 2020. Statistical analysis was performed from June 2023 to April 2024.

**Main Outcomes and Measures:**

The primary outcome was the percentage of initial opioid prescriptions filled up to 14 days prior to vs 7 days after a procedure. Secondary outcomes included the likelihood of a refill up to 180 days after surgery, including refills at 91 to 180 days, as a proxy for new persistent opioid use, and the opioid quantity dispensed in the initial and refill prescriptions in morphine milligram equivalents (MME). Exposures included patient and prescriber characteristics. Multivariable logistic regression models were used to estimate the association between prescription timing and prolonged refills.

**Results:**

Among 100 026 opioid-naive youths (median [IQR] age, 16.0 [14.0-18.0] years) undergoing a surgical procedure, 46 951 (46.9%) filled an initial prescription, of which 7587 (16.2%) were dispensed 1 to 14 days before surgery. The mean quantity dispensed was 227 (95% CI, 225-229) MME; 6467 youths (13.8%) filled a second prescription (mean MME, 239 [95% CI, 231-246]) up to 30 days after surgery, and 1216 (3.0%) refilled a prescription 91 to 180 days after surgery. Preoperative prescriptions, increasing age, and procedures not typically associated with severe pain were most strongly associated with new persistent opioid use.

**Conclusions and Relevance:**

In this retrospective study of youths undergoing surgical procedures, of which, many are typically not painful enough to require opioid use, opioid dispensing declined, but approximately 1 in 6 prescriptions were filled before surgery, and 1 in 33 adolescents filled prescriptions 91 to 180 days after surgery, consistent with new persistent opioid use. These findings should be addressed by policymakers and communicated by professional societies to clinicians who prescribe opioids.

## Key Points

**Question:**

Did pre- and postoperative opioid dispensing change between 2015 and 2020, and how often were prescriptions refilled 91 to 180 days postprocedure among opioid-naive US youths undergoing surgery?

**Findings:**

In this cohort study, the percentage of youths or caregivers who filled initial and refill prescriptions declined; however, approximately 1 in 6 youths filled their opioid prescription before surgery, and 3.0% continued to fill prescriptions 91 to 180 days after surgery.

**Meaning:**

These results suggest that although opioid prescriptions decreased over time, preoperative prescriptions and new persistent opioid use remained prevalent.

## Teaser

This cohort study investigates changes in preoperative, postoperative, and refill opioid prescriptions up to 180 days after surgery among US adolescents and young adults between 2015 and 2020.

## Introduction

Each year, approximately 1.4 million US adolescents undergo surgery.^1^ There is growing concern that filling an opioid prescription for surgical pain management may increase the risk of new persistent opioid use.^2,3^ Increased risk of adverse outcomes following opioid exposure, including development of opioid use disorder and overdose, are related to a constellation of neurobiological and social changes in adolescence.^4,5,6,7,8^ The relative strength of the adolescent brain’s dopaminergic reward system contributes to risk-taking and reward-seeking behaviors, often involving peers; meanwhile, regions of the brain such as the prefrontal cortex, which are responsible for executive function and impulse control, are relatively less well developed.^9,10,11^

One study identified that approximately 5% of opioid-naive adolescents continued to refill an opioid prescription 3 to 6 months after surgery between 2010 and 2014, compared with 0.1% of nonsurgical controls.^2^ Refills during this period are associated with persistent opioid use,^12^ which in rare cases may reflect development of an opioid use disorder.^13^ Many of these initial and refill prescriptions are not indicated^14^; in fact, professional societies and available guidance recommend nonsteroidal anti-inflammatory drugs (NSAIDs) with acetaminophen as first-line therapy for procedures that are associated with mild to moderate postoperative pain, including endoscopy and dental surgerical procedures.^15,16^

To date, little is known about initial prescription timing and refill opioid prescribing for postoperative pain management among US adolescents aged 11 to 17 years and young adults aged 18 to 20 years (hereafter, *youths*). Prior analyses have been limited geographically, often to a single facility, focused on younger children or have not included a representative range of procedures.^17,18,19,20^ In 2017, our analysis identified a decline in perioperative opioid prescribing among US youths undergoing several outpatient procedures^21^; it is unknown if this was widespread, when prescriptions were dispensed and if a decline in new persistent opioid use, defined as a refill prescription dispensed 91 to 180 days after surgery, occurred.

To address these knowledge gaps, we examined changes in the proportion of initial prescriptions that were filled up to 14 days prior to surgery, refills up to 180 days after surgery, and the quantity dispensed. Finally, we explored factors associated with new persistent opioid use. We hypothesized that preoperative prescriptions would be associated with an increased risk of prolonged opioid refills.

## Methods

### Design, Setting, and Participants

Using a national commercial insurance database, we performed a retrospective cohort study, following the Strengthening the Reporting of Observational Studies in Epidemiology (STROBE) reporting guideline,^22^ to determine perioperative opioid prescribing practices and common inpatient and outpatient procedure distribution among youths between January 1, 2015, and May 31, 2020. This study was deemed exempt from review by the University of Pennsylvania institutional review board and informed consent was waived because deidentified data were used.

We used data from Optum’s deidentified Clinformatics Data Mart Database, which contains medical and pharmacy claims data for more than 15 million annual enrollees from 50 states; adolescents are all privately insured.^23,24^ The study sample included patients aged 11 to 20 years of age on the date of surgery with a submitted claim (eFigure in Supplement 1). Because a prior analysis using some of the study data identified that procedure distribution differed between adolescents and younger children,^21^ we selected 22 procedures that are common among adolescents or considered by academic pain management clinicians to be associated with a challenging recovery. Procedures were identified using *Current Procedure Terminology (CPT)* codes (eTable 1 in Supplement 1). Individuals with more than 1 surgery on the same date or during the study period were excluded. The primary analysis included opioid-naive patients with continuous enrollment 90 days prior to surgery and 60 days after surgery. To define opioid-naive status, we selected a 90-day preoperative window based on pragmatic consensus to balance patient loss without continuous enrollment.

### Primary and Secondary Outcomes

Our primary outcome was percentage of initial opioid prescriptions filled up to 14 days prior to a procedure vs those filled up to 7 days after. Secondary outcomes included the percentage of patients who refilled a prescription up to 30 days, 31 to 60 and 91 to 180 days after surgery as a proxy for new persistent opioid use, opioid quantity dispensed, in morphine milligram equivalents (MME).

### Opioid Prescribing

In the primary analysis, we measured refills up to 60 days after surgery and 2 types of initial prescriptions: preoperative prescriptions that were filled 1 to 14 days, including 1 to 7 days and 8 to 14 days prior to surgery and postoperative prescriptions that were dispensed up to 7 days after surgery. We examined preoperative prescriptions up to 90 days before surgery and noted that an increase occurred 14 days prior to surgery, consistent with a preoperative prescription. We measured outcomes for prescriptions dispensed 30 days before surgery in a sensitivity analysis. The unanticipated proportion of preoperative prescriptions shifted the analytic focus to compare preperative vs postoperative dispensing and association with prolonged opioid refills.

Overall, 86.0% of youths (n = 40 382) had continuous enrollment 180 days after surgery through 2019 and no additional surgical procedures. We measured refills dispensed between 61 and 180 days after surgery in this secondary cohort.

We used standard conversions to calculate the quantity of opioid dispensed in MME.^25^ We included both liquid and tablet formulations of the following medications: codeine, hydrocodone, hydromorphone, morphine, oxycodone, and tramadol. For inpatient admissions, outpatient prescriptions were documented with reference to the surgery date.

### Patient and Procedure Characteristics

Demographic variables, including race and ethnicity, were provided by the claims database. We defined baseline comorbidities 90 days prior to surgery using *International Classification of Diseases, Ninth Revision (ICD-9)* and *ICD-10* diagnosis codes that were documented at least once for history of depression, anxiety, chronic pain (eTable 2 in Supplement 1), substance use disorder and overdose,^26^ and other chronic conditions categorized into 10 systems using the Pediatric Complex Chronic Conditions (CCC) classification, version 2.^27^

### Surgery Categorization

We categorized procedures by the quantity of opioid in the first prescription using historical 2010 data from the same source. Procedures were also considered from the perspective of anticipated postoperative pain (mild, moderate, or severe) based upon expert consensus and pediatric guidelines due to differences between anticipated pain and opioid dispensing.^15,16^ Procedures were analyzed in 3 groups: (1) higher prescribing: spinal fusion, craniotomy, Nuss bar procedures, knee arthroscopy, tonsillectomy, lower extremity fracture, colectomy, and bariatric surgery; (2) medium prescribing: exploratory laparotomy, Le Fort procedures, laparoscopic cholecystectomy, rhinoplasty, hardware removal, dental surgery, and breast surgery; and (3) lower prescribing: appendectomy, incision and drainage, endoscopy, orchiopexy, tympanoplasty, circumcision, and supracondylar fracture repair.

### Statistical Analysis

#### Primary and Secondary Analysis

We compared procedures and patient characteristics by prescribing category and age groups (11-17 years and 18-20 years), defined a priori with American Academy of Pediatrics guidance.^28^ We used simple hypothesis tests and descriptive figures to characterize outcomes, including 60-day refills. The secondary analysis used the same methods to characterize opioid prescriptions dispensed 61 to 180 days after surgery among youths with continuous enrollment.

#### Regression Models

We built multivariable logistic regression models to estimate the association between procedural and patient characteristics and an opioid prescription refill during 3 previously described periods. The model measured the adjusted and unadjusted association of the procedure, year of surgery and specific patient factors, including age, modeled as independent variables by year with age 11 years as the referent, depression, anxiety, chronic pain diagnosis, substance use disorder history,^26^ and timing of the initial prescription on the odds of refills. Knee arthroscopy, the reference procedure, was common with an average percentage of refills. Due to visible trend changes by year, each year was an independent variable with 2015 as the referent. We also performed a sensitivity regression analysis to examine the association of MME dose ranges (≤25 MME, 26-60, 61-100, 101-200, >200 MME) with refills. Analyses were conducted in SAS version 9.4 (SAS Institute) from June 2023 to April 2024. All tests were 2-sided and significance was set at the 5% level.

## Results

### Descriptive Data

The cohort, described in Table 1,^29^ included 100 026 opioid-naive youths, with a median (IQR) age of 16.0 (14.0-18.0) years, who underwent a surgical procedure between 2015 and 2020; 49 580 (49.6%) were female, 50 445 (50.4%) were male, 7104 (7.1%) were African American or Black, 2949 (2.9%) were Asian, 11 099 (11.1%) were Hispanic or Latino, 69 649 (69.6%) were White, and 9225 (9.2%) were other race or ethnicity; 46 951 youths (46.9%) filled an opioid prescription, of which 16.2% (7587 of 46 951) were dispensed 1 to 14 days prior to the procedure. The mean MME dispensed was 227 (95% CI, 225-229) (Table 2). Within surgical dispensing categories, there were increases in prescriptions, the proportion filled before surgery, and quantity dispensed among older youths aged 18 to 20 years. Table 3 displays refill prescription characteristics at 3 time points. Among those dispensed an initial opioid prescription, 6467 (13.8%) filled a second prescription within 30 days of surgery that contained a mean MME of 239 (95% CI, 231-246). Between 31 and 60 days after surgery, 685 youths (1.5%) filled a prescription with a mean MME of 218 (95% CI, 199-238). Among patients with continuous enrollment 91 to 180 days after surgery, 1216 (3.0%) filled an additional prescription with a mean MME of 155 (95% CI, 146-164).

**Table 1.** Patient Demographics Among Opioid-Naive Youths With Continuous Enrollment, 2015-2020

Patient characteristics	Age 11-17 y (n = 64 711)	Age 18-20 y (n = 35 315)	Overall (n = 100 026)
Age, median (IQR), y	15.0 (13.0-16.0)	19.0 (18.0-20.0)	16.0 (14.0-18.0)
Sex, No. (%)			
Female^a^	31 098 (48.1)	18 482 (52.3)	49 580 (49.6)
Male	33 613 (51.9)	16 832 (47.7)	50 445 (50.4)
Race and ethnicity			
African American/Black	4340 (6.7)	2764 (7.8)	7104 (7.1)
Asian	2039 (3.2)	910 (2.6)	2949 (2.9)
Hispanic/Latino	7230 (11.2)	3869 (11.0)	11 099 (11.1)
White, non-Hispanic	45 004 (69.5)	24 645 (69.8)	69 649 (69.6)
Other/unknown^b^	6098 (9.4)	3127 (8.9)	9225 (9.2)
Outpatient procedure, No. (%)	58 813 (90.9)	32 726 (92.7)	91 539 (91.5)
Common comorbidities, No. (%)			
Congenital defect diagnosis	2803 (4.3)	813 (2.3)	3616 (3.6)
Cardiovascular diagnosis	1262 (2.0)	683 (1.9)	1945 (1.9)
Neurologic/neuromuscular diagnosis	1416 (2.2)	537 (1.5)	1953 (2.0)
Malignancy diagnosis	1048 (1.6)	652 (1.8)	1700 (1.7)
Gastrointestinal diagnosis	1198 (1.9)	1070 (3.0)	2268 (2.3)
Depression	739 (1.1)	671 (1.9)	1410 (1.4)
Anxiety	3910 (6.0)	2702 (7.7)	6612 (6.6)
History of substance use disorder	104 (0.2)	258 (0.7)	362 (0.4)
History of overdose	32 (>0.1)	34 (0.1)	66 (0.1)
Chronic pain diagnosis	4886 (7.6)	3347 (9.5)	8233 (8.2)

^a^ Sex was missing for 1 patient.

^b^ The definition of other race is defined internally by Optum DataMart using a proprietary unpublished algorithm.^29^

**Table 2.** Preoperative and Postoperative Initial Opioid Prescriptions Among Opioid-Naive Patients With Continuous Enrollment, 2015-2020

Variables	High prescribing^a^		Medium prescribing		Low prescribing		Overall (n = 100 026)
	11-17 y (n = 17 119)	18-20 y (n = 7661)	11-17 y (n = 15 094)	18-20 y (n = 8946)	11-17 y (n = 32 498)	18-20 y (n = 18 708)	
Filled opioid prescription, No. (%)	12 173 (71.1)	5656 (73.8)	9216 (61.)	6249 (69.9)	8455 (26.0)	5202 (27.8)	46 951 (46.9)
Mean quantity, MME (95% CI)	296 (291-300)	326 (319-333)	227 (222-231)	226 (221-232)	116 (114-118)	142 (140-145)	227 (225-229)
% Of prescriptions filled pre-operatively, No. (%)	2191 (18.0)	1229 (21.7)	1442 (15.6)	1365 (21.8)	744 (8.8)	616 (11.8)	7587 (16.2)

^a^ High-prescribing procedures include spinal fusion, craniotomy, Nuss bar procedures, knee arthroscopy, tonsillectomy, lower extremity fracture, colectomy and bariatric surgery; medium-prescribing procedures included exploratory laparotomy, Le Fort procedures, laparoscopic cholecystectomy, rhinoplasty, hardware removal, dental surgery and breast surgery; lower-prescribing procedures include appendectomy, incision and drainage, endoscopy, orchiopexy, tympanoplasty, circumcision, and supracondylar fracture repair.

**Table 3.** Refill Opioid Prescriptions After Surgery Among Opioid-Naive Patients With Continuous Enrollment, 2015-2020

Variables	High prescribing		Medium prescribing		Low prescribing		Overall (n = 46 951)
	11-17 y (n = 12 173)	18-20 y (n = 5656)	11-17 y (n = 9216)	18-20 y (n = 6249)	11-17 y (n = 8455)	18-20 y (n = 5502)	
Refill dispensed up to 30 d after surgery, No. (%)	2087 (17.1)	1321 (23.4)	1099 (11.9)	1001 (16.0)	462 (5.5)	497 (9.6)	6467 (13.8)
Mean quantity, MME (95% CI)	283 (267-300)	309 (286-332)	181 (170-192)	208 (195-221)	178 (162-195)	177 (158-196)	239 (231-246)
Refill dispensed 31-60 d after surgery, No. (%)	171 (1.4)	132 (2.3)	73 (0.8)	85 (1.4)	87 (1.0)	137 (2.6)	685 (1.5)
Mean quantity, MME (95% CI)	234 (200-267)	315 (251-379)	161 (131-190)	175 (142-207)	182 (110-254)	187 (155-219)	218 (199-238)
Refill dispensed 91-180 d after surgery, No. (%)^a^	274 (2.6)	163 (3.4)	213 (2.6)	207 (3.8)	156 (2.2)	203 (4.6)	1216 (3.0)
Mean quantity, MME (95% CI)	174 (156-192)	202 (167-238)	137 (121-153)	133 (117-150)	145 (117-173)	141 (124-157)	155 (146-164)

^a^ Denominator is number of patients with no additional surgeries who had continuous enrollment up to 180 days after surgery (n = 40 382) and filled an initial prescription for surgery.

### Opioid Prescribing Characteristics by Year, Timing of Initial Prescription, and Surgery

**Figure 1.**
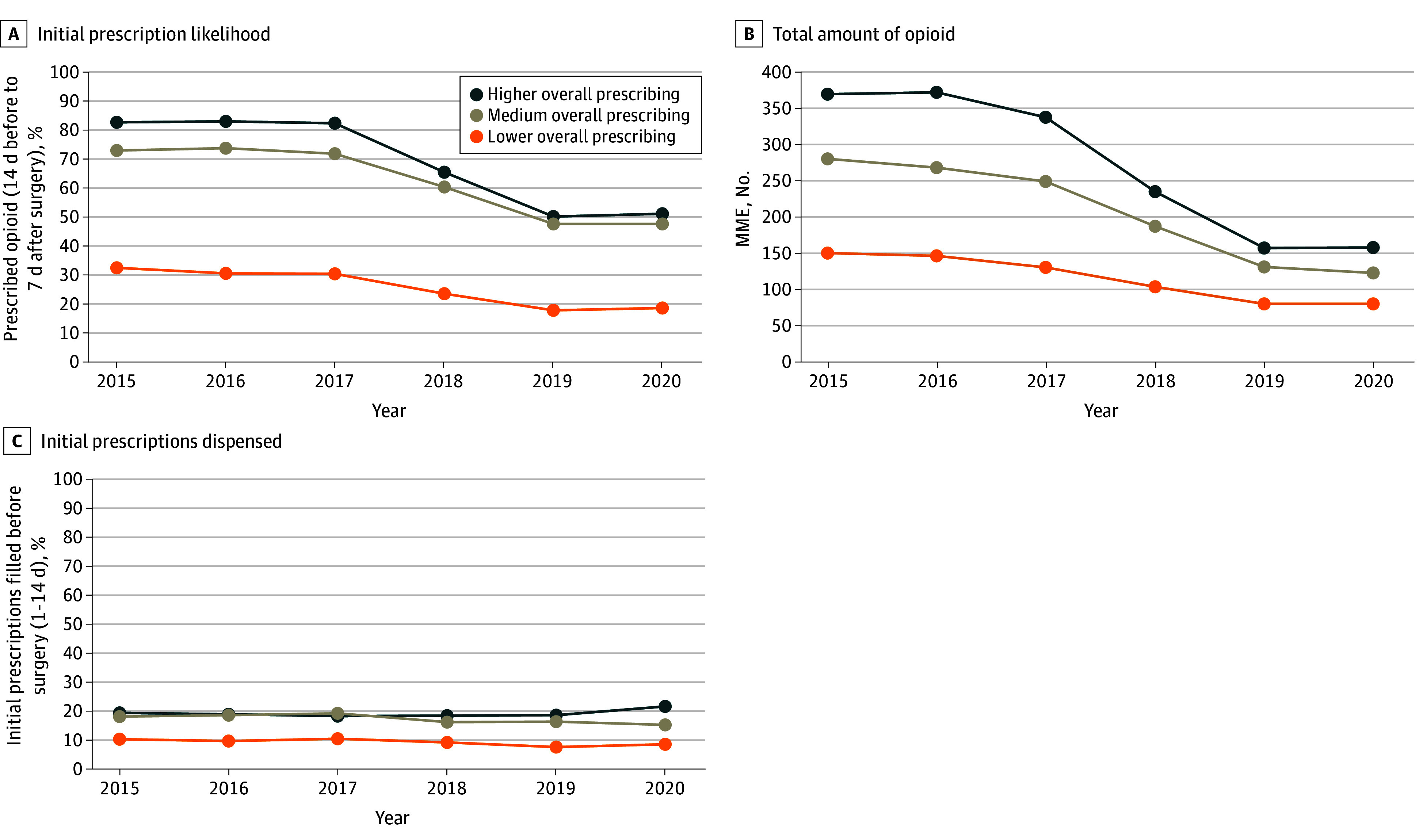
Characteristics of Initial Preoperative and Postoperative Opioid Prescriptions, 2015-2020 By year, each figure displays characteristics of initial opioid prescriptions filled between 14 days prior to and up to 7 days after surgery among opioid-naive youths undergoing study procedures. Procedures are grouped by higher, medium, and lower overall opioid-prescribing categories. Panels show initial prescription likelihood (A), total amount of opioid in MME dispensed in the first prescription (B), and percentage of initial prescriptions dispensed up to 14 days prior to surgery (C). MME indicates morphine milligram equivalents.

**Figure 2.**
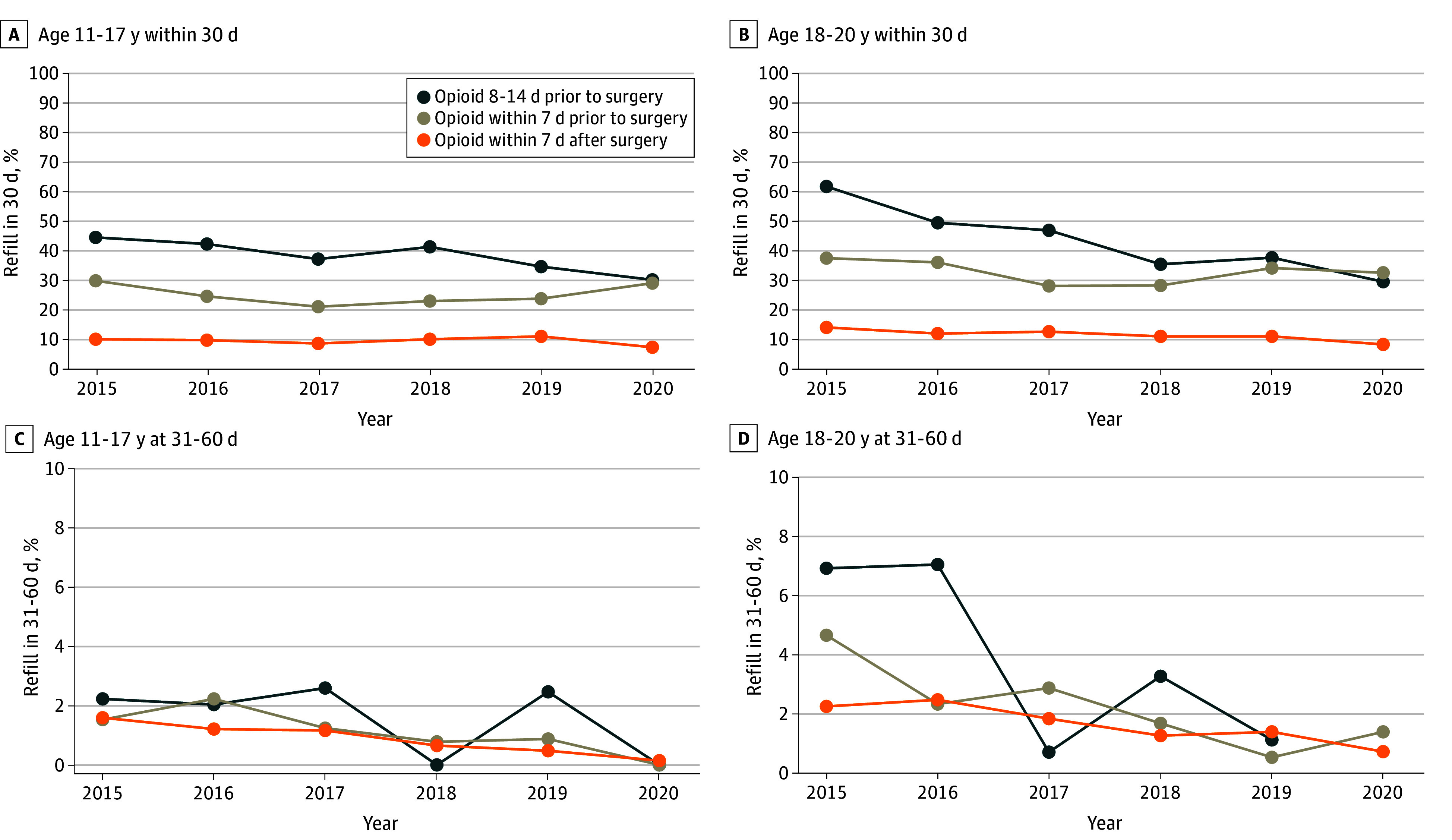
Percentage of Opioid-Naive Youths Who Obtained an Opioid Prescription Refill Up to 60 Days After Surgery, by Timing of First Opioid Prescription Fill By year, each panel displays the percentage of opioid-naive youths with continuous enrollment 90 days prior to and 60 days after a given surgery who were dispensed a refill opioid prescription within 30 days after surgery, by timing of first opioid prescription fill. A, The percentage of youths aged 11 to 17 years who filled a refill within 30 days after surgery. B, The percentage of youths aged 18 to 20 years who filled a refill within 30 days after surgery. C, The percentage of youths aged 11 to 17 years who filled a refill prescription 31 to 60 days after surgery. D, The percentage of youths aged 18 to 20 years who filled a refill prescription 31 to 60 days after surgery. The 3 lines in each panel refer to the time of the first opioid prescription with regard to surgery: 14 to 8 days prior, 7 to 1 days prior, and within 1 week of surgery.

The likelihood of filling an index opioid prescription, mean MME dispensed, and proportion of initial prescriptions dispensed before surgery differed between prescribing categories (Figure 1). The proportion of filled prescriptions and quantity dispensed declined beginning in 2017, but the proportion of preoperative fills remained similar. We also examined these practices by procedure and age group (eTable 3 in Supplement 1). Preoperative prescriptions were more common among ages 18 to 20 years and were filled for procedures unlikely to be associated with severe preoperative pain, including breast surgery, dental procedures, and rhinoplasty. Figure 2 displays preoperative prescription timing vs percentage of patients obtaining an initial refill up to 30 days and 31 to 60 days after surgery. The overall likelihood of filling a second prescription was elevated if the first prescription was filled between 8 and 14 days and 1 and 7 days prior to surgery. Findings were similar in the sensitivity analysis that included youths with prescriptions dispensed up to 30 days prior to surgery (eTable 4 in Supplement 1).

The percentage of patients who received a preoperative opioid prescription appeared stable between 2015 and 2020 (eTable 5 in Supplement 1). However, the percentage of 30-day refills appeared to decline, from 15.5% (1513 of 9731) to 11.2% (145 of 1292), and the mean MME dispensed appeared to decline, from 264 (95% CI, 248-280) to 199 (95% CI, 161-238). As a proxy for new persistent use, prescriptions dispensed 91 to 180 days after surgery declined from 3.5% (306 of 8720) in 2015 to 2.1% (122 of 5747) in 2019, the last year with full data for this period. The MME dispensed declined from 184 (95% CI, 160-207) to 110 (95% CI, 98-122).

### Factors Associated With New Persistent Opioid Use

Three multivariable logistic regression analyses identified associations between patient and procedural factors and the odds of opioid prescription refills 1 to 30 days, 31 to 60 days, and 91 to 180 days after surgery (eTable 6 in Supplement 1). Characteristics most strongly associated with refill up to 30 days after surgery included opioid prescriptions filled 8 to 14 days prior to surgery (adjusted odds ratio, 5.98 [95% CI, 5.27-6.78]) compared with filling after surgery and increasing age (adjusted odds ratio for aged 20 years vs aged 11 years: 2.72 [95% CI, 2.34-3.16]). With regard to prescription refills 91 to 180 days after surgery, filling a prescription before surgery and each year of increasing age was associated with a similar increase in risk. In the sensitivity analysis that included MME dose ranges, the 91- to 180-day refills did not have a consistent association with initial prescription MME, while both lower and higher initial MMEs were associated with increased refills up to 30 days in the unadjusted analysis, with a significant interaction between surgery type and initial MME dispensed in the adjusted analysis (*P* < .001) (eTable 7 in Supplement 1).

## Discussion

Among 100 026 youths aged 11 to 20 years undergoing 22 common procedures between 2015 and 2020, we observed a high proportion of preoperative opioid prescriptions and opioid doses that often contradicted professional recommendations.^15,16^ We observed that 3.0% of youths with continuous enrollment continued to fill prescriptions between 91 and 180 days after surgery, as a proxy for new persistent opioid use.^12^ Despite decreases in filled prescription and quantity dispensed over time, these high-risk prescribing practices remained common at the end of the study period. We also noted increases in the proportion filling prescriptions, quantity dispensed, and prolonged refills among older youths aged 18 to 20 years compared with younger youths, which may be related to care by adult clinicians, where similar trends have been identified, or to the youth’s decision-making status as a legal adult.^30,31^ We identified patient and procedural/prescription characteristics that were associated with prolonged refills, including increasing age, female gender, a chronic pain diagnosis, and filling a preoperative opioid prescription for a procedure not typically associated with severe preoperative pain. Of note, initial opioid prescriptions for procedures with severe postoperative pain were not associated with an increased risk of new persistent opioid use, suggesting that severity of acute postoperative pain may not be a risk factor for prolonged refills. Increased opioid exposure, with amount of time dispensed prior to surgery and total MME as proxies, have been associated with new persistent opioid use in the adult literature.^30,31^ Our findings also support an identified association between preexisting pain disorders, psychiatric comorbidities, and risk of prolonged opioid fills, which may be suggestive of attempts to treat nonsurgical pain or self-manage psychiatric symptoms.^31^

Prescriptions were dispensed up to 2 weeks prior to surgery for procedures unlikely to be associated with severe preoperative pain, including dental and plastic surgical procedures, and preoperative dispensing was observed to correlate with an increased odds of obtaining refill prescriptions throughout the study period. In addition, quantities were often in excess of recommended prescribing guidelines, raising concerns of increased community supply.^15,16,32^ However, patient- or procedure-specific considerations should remain an important consideration as surgeons are encouraged to follow guideline recommendations. The 2 national resources specific to pediatric surgery were both published in 2020 at the end of the study period and include the Michigan OPEN opioid prescribing recommendations and expert consensus guidelines for recommended vs possible opioid-free recoveries.^15,16^ Michigan OPEN offers weight-based guidance for adolescents and advises no outpatient opioids for procedures including appendectomy, tympanoplasty and orthopedic hardware removal and a limited course of opioids for more painful procedures (ie, a maximum of 14 outpatient doses for knee arthroscopy).^15^ Recommendations are supported by studies, including one that interviewed pediatric patients after appendectomy and identified the majority of patients did not need any opioids and a maximum of 3 doses (<25 MME) was appropriate for the small subset of patients who reported opioid consumption.^33^ In contrast, a mean of 118 MME and 160 MME were dispensed nationally after appendectomy to younger and older youths, respectively.

Factors associated with 30-day and 91- to 180-day refills differed slightly. Overall, a constellation of characteristics, including preoperative prescriptions, increasing age, a history of anxiety, functional chronic pain, and female sex were consistently observed risk factors. Preoperative prescriptions, increasing age, orthopedic procedures, dental surgery, tonsillectomy, history of substance use disorder and overdose were most strongly associated with opioid prescription refills up to 30 days after surgery. Interestingly, we did not observe an association between undergoing a procedure commonly associated with severe postoperative pain or a procedure where preoperative opioids may be indicated and persistent opioid use risk.

Our study filled several important knowledge gaps with regard to perioperative pain management. First, we established the proportion of youths age 11 to 20 years who filled preoperative prescriptions and initial prescription characteristics for common procedures between 2015 and 2020. Next, we demonstrated that the percentage of patients who received initial and refill prescriptions and the opioid quantity dispensed decreased over the study period. This is likely related to increased clinician awareness of appropriate surgery-specific opioid dosing guidelines and national concern over the risks associated with excess opioid supply in the community.^4,15,20^ We also identified that new persistent opioid use declined over time, from 3.5% to 2.1%, possibly as a result of the observed decline in initial opioid prescriptions.

### Limitations

This study has limitations. First, patient data were obtained from a private insurance claims database, and findings may not be generalizable to youths with public insurance coverage or those without insurance. The population also underrepresented youths of Black or African American race and Hispanic or Latino ethnicity, which could introduce unintended selection bias. Medical visits, comorbidity diagnosis, and prescription fills not associated with a patient’s insurance were not captured in this analysis. In addition, we studied opioid prescription refills, not self-reported consumption. Individuals who are considered to be opioid-naive may have had opioid exposures prior to the 90-day continuous enrollment screening period. It is also possible that refills were not consumed by the youth and were unused or possibly diverted. Despite a decrease in initial prescriptions over time, it is notable that refill prevalence did not increase, suggesting that pain was adequately treated without these opioid prescriptions, and that prescription quantities at the end of the study period were often in excess of guideline recommendations. Increasing age had a strong association with refills; however, we were unable to determine if the index prescriber was a primary pediatric or adult clinician to determine if this influenced management of older patients. Because we were unable to track prescribers or determine if youths were obtaining new prescriptions rather than refilling the original prescription, it was reassuring that prescription monitoring programs were commonly used during the study period to decrease the risk of duplicate prescriptions.^34^

## Conclusions

This cohort study identified concerning outcomes, including routine preoperative opioid prescriptions and prolonged refills despite an overall decline in both initial and refill prescriptions. Opioid prescriptions and quantities dispensed were often inconsistent with professional society or guideline recommendations. At the end of the study period, 2.1% of youths experienced new persistent opioid use 3 to 6 months following surgery. While this represents a steady decline over time, it is still cause for action. Youths who underwent a procedure that is typically associated with severe pain or one considered an indication for preoperative opioid use were not observed to have an increased risk of new persistent opioid use. These findings should prompt policymakers and perioperative prescribers to use available opioid prescribing guidelines as the standard of care for youths who may require an opioid prescription for pain management.

## Notes

Supplement 1. **eFigure.** Inclusion and Exclusion Criteria for Primary and Secondary Study Cohort **eTable 1.** CPT Procedure Codes **eTable 2.** ICD Codes for Comorbidities **eTable 3.** Initial Opioid Prescriptions for Opioid-Naïve Patients, by Procedure, 2015-2020 **eTable 4.** Initial and Refill Opioid Prescriptions After Surgery Among Opioid-Naïve Patients, Including Those Who Filled Prescriptions Up to 30 Days Prior to Surgery, 2015-2020 **eTable 5.** Preoperative and Refill Opioid Prescription Trends Among Patients Who Received an Initial Prescription, by Year **eTable 6.** Adjusted and Unadjusted Odds Ratios for Likelihood of Prescription Refill at Three Intervals **eTable 7.** Adjusted and Unadjusted Odds Ratios for Likelihood of Prescription Refill at Three Intervals, Sensitivity Analysis with MME Ranges

Supplement 2.
Data Sharing Statement
