# Supplementary material for: Preoperative vs Postoperative Opioid Prescriptions and Prolonged Opioid Refills Among US Youths
Source: JAMA Netw Open. 2024 Jul 5;7(7):e2420370. doi: 10.1001/jamanetworkopen.2024.20370 (PMC11227082; doi:10.1001/jamanetworkopen.2024.20370)
Supplement: Supplement 1. — eFigure. Inclusion and Exclusion Criteria for Primary and Secondary Study Cohort eTable 1. CPT Procedure Codes eTable 2. ICD Codes for Comorbidities eTable 3. Initial Opioid Prescriptions for Opioid-Naïve Patients, by Procedure, 2015-2020 eTable 4. Initial and Refill Opioid Prescriptions After Surgery Among Opioid-Naïve Patients, Including Those Who Filled Prescriptions Up to 30 Days Prior to Surgery, 2015-2020 eTable 5. Preoperative and Refill Opioid Prescription Trends Among Patients Who Received an Initial Prescription, by Year eTable 6. Adjusted and Unadjusted Odds Ratios for Likelihood of Prescription Refill at Three Intervals eTable 7. Adjusted and Unadjusted Odds Ratios for Likelihood of Prescription Refill at Three Intervals, Sensitivity Analysis with MME Ranges [file jamanetwopen-e2420370-s001.pdf]

Supplemental Online Content

Sutherland TN, Rabbitts JA, Tasian GE, Neuman MD, Newcomb C, Hadland SE. Preoperative vs postoperative opioid prescriptions and prolonged opioid refills among US youths. *JAMA Netw Open.* 2024;7(7):e2420370. doi:10.1001/jamanetworkopen.2024.20370

eFigure. Inclusion and Exclusion Criteria for Primary and Secondary Study Cohort

eTable 1. CPT Procedure Codes

eTable 2. ICD Codes for Comorbidities

eTable 3. Initial Opioid Prescriptions for Opioid-Naïve Patients, by Procedure, 2015-2020

eTable 4. Initial and Refill Opioid Prescriptions After Surgery Among Opioid-Naïve Patients, Including Those Who Filled Prescriptions Up to 30 Days Prior to Surgery, 2015-2020

eTable 5. Preoperative and Refill Opioid Prescription Trends Among Patients Who Received an Initial Prescription, by Year

eTable 6. Adjusted and Unadjusted Odds Ratios for Likelihood of Prescription Refill at Three Intervals

eTable 7. Adjusted and Unadjusted Odds Ratios for Likelihood of Prescription Refill at Three Intervals, Sensitivity Analysis with MME Ranges

This supplemental material has been provided by the authors to give readers additional information about their work.

© 2024 Sutherland TN et al. *JAMA Network Open.*

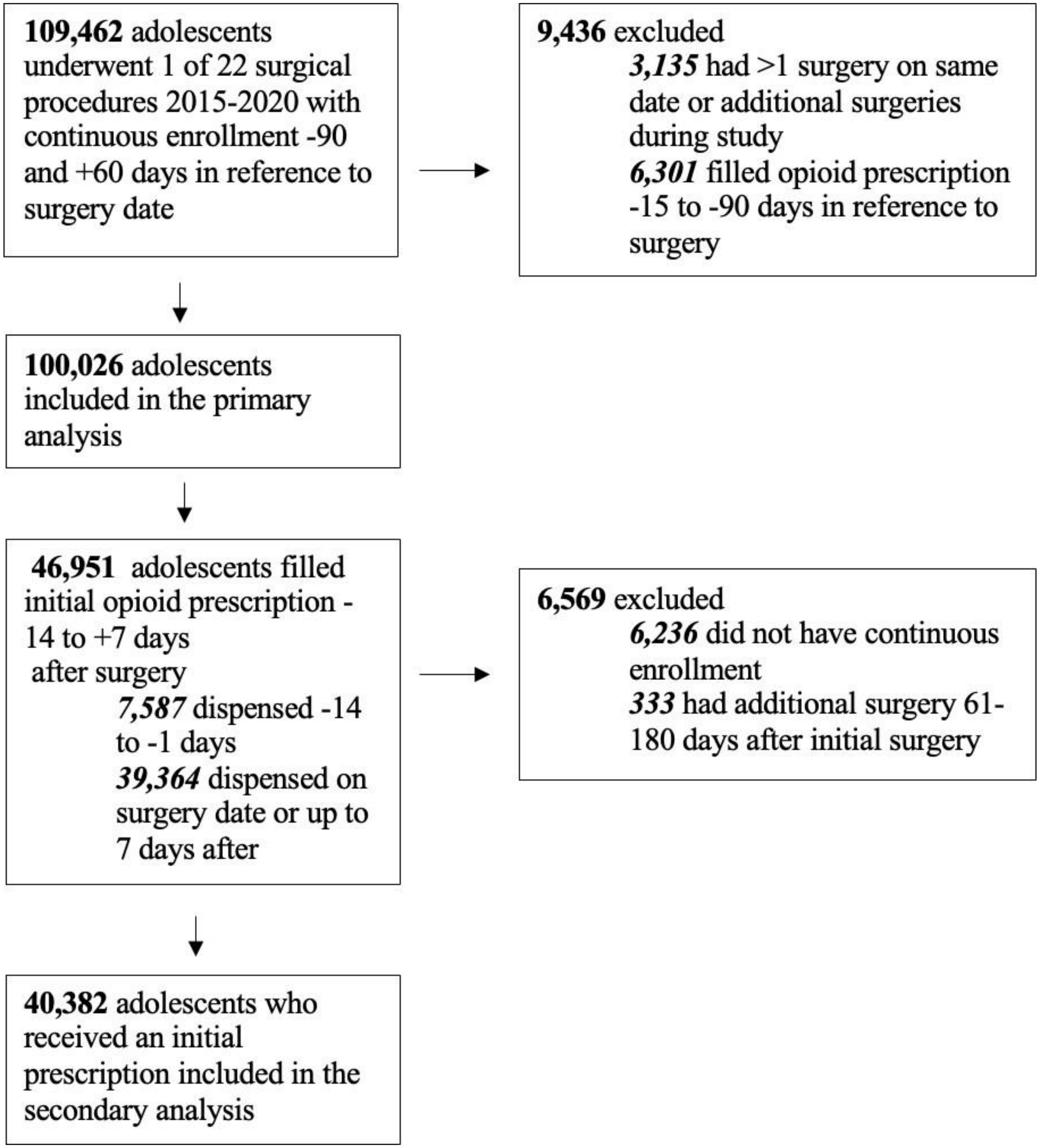

eFigure 1. Inclusion and Exclusion criteria for Primary and Secondary Study Cohorts

© 2024 Sutherland TN et al. *JAMA Network Open.*

eTable 1. CPT Procedure Codes	
**Procedure name**	**CPT Code**
**Appendectomy**	44970
**Bariatric procedures (lap band, sleeve)**	43842, 43843, 43845-48, 43644, 43645, 43659, 43770, 43775, 43770, 43843
**Breast reduction/augmentation**	19325, 19318
**Circumcision**	54150-54163
**Colectomy**	44140, 44141, 44143, 44144, 44145, 44146, 44147, 44204, 44205, 44206, 44207, 44208, 44160
**Craniotomy**	61304-61576
**Dental surgery**	41899
**Endoscopy/colonoscopy**	43200-02, 43215; 45378
**Exploratory laparatomy**	44120, 49000
**Hardware removal**	20670, 20680
**Incision and drainage of wound**	10060-61, 10140, 10180, 20005, 23930, 23931, 25028, 25035, 11010-12
**Knee arthroscopy**	29881; 29880; 29877; 29875; 29876; 29870
**Laparoscopic cholecystectomy**	47562; 47563; 47564
**Le Fort/Craniofacial**	21141-47; 21150-51; 21193-96; 21208, 21209
**Lower extremity fracture repair**	27758, 27759, 27720, 27722, 27724, 27511, 27513, 27766, 27769, 27832, 27829
**Nuss bar insertion**	21742-43
**Orchiopexy**	54640, 54600
**Rhinoplasty**	30400, 30410, 30420, 20460, 30462, 30465
**Spinal fusion**	22612, 22551, 22554, 22610, 22600, 22556, 22558, 22800, 22802, 22804, 22849, 22808, 22806, 22812
**Supracondylar fracture repair**	24538, 24545, 24546, 24566, 24575, 24582
**Tonsillectomy and/or adenoidectomy**	42820, 42825, 42821, 42826
**Tympanoplasty**	69620, 69631, 69632, 69633, 69635, 69636, 69637, 69641, 69642, 69643, 69644, 69645, 69646

© 2024 Sutherland TN et al. *JAMA Network Open.*

eTable 2. ICD Codes for Comorbidities		
**Diagnoses**	**ICD-9**	**ICD-10**
**Depression**	296.2, 296.21, 296.22, 296.23, 296.24, 296.25, 296.26	F33.0, F33.1, F33.2
**Anxiety**	300.1, 300.02	F41.1 F41.9, F43.23
**History of Chronic Pain**	338.21, 338.22, 338.28, 338.29, 338.3, 353.6, 337.22, 355.71	G89.2, F45.41, M54, R51.9, G90.5, G90.9, M79.1, M79.7 T82.84, T83.84, T84.84, R52.1, R52.2, G44.32, G44.04, G44.02, G43.7

© 2024 Sutherland TN et al. *JAMA Network Open.*

eTable 3. Initial opioid prescriptions for opioid-naïve patients, by procedure, 2015-2020									
**Procedure**	**Age 11-17**				**Age 18-20**				**Overall**
	**Number of patients, n(%)**	**Filled opioid prescription, n(%)**	**Average Quantity, in MME**	**Prescription filled pre-operatively, n(%)**	**Number of patients, n(%)**	**Filled opioid prescription, n(%)**	**Average Quantity, in MME**	**Prescription filled pre-operatively, n(%)**	
**High-Prescribing Procedures**									
Bariatric surgery	49 (0.1%)	26 (53.1%)	153 (109 - 197)	3 (6.1%)	141 (0.4%)	101 (71.6%)	180 (155 - 204)	38 (27.0%)	190 (0.2%)
Colectomy, partial or complete	135 (0.2%)	61 (45.2%)	189 (129 - 248)	7 (5.2%)	109 (0.3%)	66 (60.6%)	217 (170 - 264)	3 (2.8%)	244 (0.2%)
Craniotomy	530 (0.8%)	229 (43.2%)	202 (175 - 228)	24 (4.5%)	222 (0.6%)	115 (51.8%)	334 (280 - 389)	10 (4.5%)	752 (0.8%)
Knee arthroscopy	7,351 (11.4%)	5,311 (72.2%)	262 (257 - 266)	883 (12.0%)	4,180 (11.8%)	3,025 (72.4%)	289 (282 - 296)	548 (13.1%)	11,531 (11.5%)
Lower extremity fracture repair	1,190 (1.8%)	875 (73.5%)	219 (206 - 232)	378 (31.8%)	627 (1.8%)	468 (74.6%)	264 (243 - 286)	225 (35.9%)	1,817 (1.8%)
Nuss bar insertion	323 (0.5%)	229 (70.9%)	323 (290 - 356)	50 (15.5%)	41 (0.1%)	33 (80.5%)	545 (339 - 750)	13 (31.7%)	364 (0.4%)
Spinal fusion	1,619 (2.5%)	1,132 (69.9%)	419 (399 - 438)	231 (14.3%)	291 (0.8%)	194 (66.7%)	497 (437 - 557)	41 (14.1%)	1,910 (1.9%)
Tonsillectomy	5,922 (9.2%)	4,310 (72.8%)	326 (319 - 334)	615 (10.4%)	2,050 (5.8%)	1,654 (80.7%)	399 (383 - 415)	351 (17.1%)	7,972 (8.0%)
**Medium-Prescribing Procedures**									
Breast surgery	219 (0.3%)	163 (74.4%)	189 (171 - 208)	83 (37.9%)	476 (1.3%)	343 (72.1%)	214 (201 - 228)	141 (29.6%)	695 (0.7%)
Cholecystectomy	1,539 (2.4%)	1,031 (67.0%)	138 (133 - 143)	140 (9.1%)	2,174 (6.2%)	1,557 (71.6%)	163 (158 - 168)	267 (12.3%)	3,713 (3.7%)
Le Fort/ Craniofacial surgery	345 (0.5%)	245 (71.0%)	164 (151 - 178)	89 (25.8%)	352 (1.0%)	268 (76.1%)	174 (160 - 187)	100 (28.4%)	697 (0.7%)
Dental surgery	8,491 (13.1%)	5,445 (64.1%)	272 (265 - 279)	825 (9.7%)	3,616 (10.2%)	2,645 (73.1%)	282 (270 - 293)	539 (14.9%)	12,107 (12.1%)
Exploratory laparotomy	97 (0.1%)	39 (40.2%)	171 (131 - 212)	5 (5.2%)	94 (0.3%)	60 (63.8%)	207 (168 - 246)	7 (7.4%)	191 (0.2%)
Hardware removal	3,831 (5.9%)	1,887 (49.3%)	170 (162 - 177)	179 (4.7%)	1,588 (4.5%)	912 (57.4%)	216 (204 - 228)	121 (7.6%)	5,419 (5.4%)
Rhinoplasty	572 (0.9%)	406 (71.0%)	165 (156 - 174)	121 (21.2%)	646 (1.8%)	464 (71.8%)	184 (174 - 194)	190 (29.4%)	1,218 (1.2%)
**Low-Prescribing Procedures**									
Appendectomy, laparoscopic or open	7,884 (12.2%)	3,908 (49.6%)	118 (116 - 121)	101 (1.3%)	3,792 (10.7%)	2,585 (68.2%)	160 (156 - 163)	89 (2.3%)	11,676 (11.7%)
Circumcision	1,289 (2.0%)	698 (54.2%)	94 (90 - 98)	28 (2.2%)	425 (1.2%)	223 (52.5%)	140 (129 - 152)	12 (2.8%)	1,714 (1.7%)

© 2024 Sutherland TN et al. *JAMA Network Open.*

Endoscopy	696 (1.1%)	21 (3.0%)	119 (84 - 154)	10 (1.4%)	2,177 (6.2%)	56 (2.6%)	142 (115 - 169)	43 (2.0%)	2,873 (2.9%)
Incision and drainage	18,775 (29.0%)	1,530 (8.1%)	116 (109 - 123)	288 (1.5%)	11,701 (33.1%)	1,923 (16.4%)	117 (111 - 122)	413 (3.5%)	30,476 (30.5%)
Orchiopexy	1,484 (2.3%)	968 (65.2%)	98 (94 - 102)	27 (1.8%)	199 (0.6%)	143 (71.9%)	134 (122 - 145)	10 (5.0%)	1,683 (1.7%)
Supracondylar fracture repair	711 (1.1%)	547 (76.9%)	152 (141 - 163)	180 (25.3%)	38 (0.1%)	32 (84.2%)	278 (194 - 363)	15 (39.5%)	749 (0.7%)
Tympanoplasty	1,659 (2.6%)	783 (47.2%)	125 (120 - 130)	110 (6.6%)	376 (1.1%)	240 (63.8%)	154 (144 - 165)	34 (9.0%)	2,035 (2.0%)
OVERALL	N=64,711	29,844 (46.1%)	223 (221 - 226)	4,377 (6.8%)	N=35,315	17,107(48.4%)	234 (230- 237)	3,210 (9.1%)	N=100,026

© 2024 Sutherland TN et al. *JAMA Network Open.*

**eTable 4. Initial and refill opioid prescriptions after surgery among opioid-naïve patients, including those who filled prescriptions up to 30 days prior to surgery, 2015-2020**							
	**High Prescribing**		**Medium Prescribing**		**Low Prescribing**		**Overall**
Filled initial opioid prescription, N	11-17 years (N= 17,361)	18-20 years (N= 7,843)	11-17 years (N= 15,561)	18-20 years (N= 9,280)	11-17 years (N= 36,662)	18-20 years (N= 18,872)	N= 101,579
Filled initial opioid prescription, n (%)	12,415	5,838	9,683	6,583	8,619	5,366	48,504
Average quantity, in MME (95% CI)	293 (289 - 297)	321 (314 - 328)	223 (219 - 227)	223 (218 - 228)	116 (114 - 118)	142 (139 - 145)	225 (223 - 227)
Percentage of prescriptions filled pre-operatively, n(%)	2,433 (19.6%)	1,411 (24.2%)	1,909 (19.7%)	1,699 (25.8%)	908 (10.5%)	780 (14.5%)	9,140 (18.8%)
Refill dispensed up to 30 days after surgery, n (%)	2,143 (17.3%)	1,378 (23.6%)	1,117 (11.5%)	1,049 (15.9%)	471 (5.5%)	521 (9.7%)	6,679 (13.8%)
Average quantity, in MME (95% CI)	288 (271 - 305)	311 (288 - 333)	183 (171 - 194)	210 (197 - 223)	179 (163 - 195)	177 (158 - 195)	241 (234 - 249)
Refill dispensed 31-60 days after surgery, n (%)	180 (1.4%)	143 (2.4%)	84 (0.9%)	95 (1.4%)	93 (1.1%)	154 (2.9%)	749 (1.5%)
Average quantity, in MME (95% CI)	235 (202 - 268)	304 (245 - 363)	163 (135 - 190)	175 (145 - 205)	182 (114 - 249)	193 (163 - 223)	217 (199 - 236)
Refill dispensed 91-180 days after surgery, n (%)[1]	288 (2.7%)	173 (3.5%)	229 (2.7%)	229 (4.0%)	161 (2.2%)	215 (4.8%)	1,295 (3.1%)
Average quantity, in MME (95% CI)	177 (159 - 195)	200 (166 - 234)	140 (125 - 155)	136 (121 - 152)	147 (119 - 175)	151 (128 - 173)	158 (150 - 167)

1.Denominator is number of patients with no additional surgeries who had continuous enrollment up to 180 days after surgery (n=40,382) and filled an initial prescription for surgery.

© 2024 Sutherland TN et al. *JAMA Network Open.*

eTable 5. Preoperative and refill opioid prescription trends among patients who received an initial prescription, by year							
	**2015**	**2016**	**2017**	**2018**	**2019**	**2020[1]**	**Overall**
**Patients, N**	9,731	10,376	10,584	8,431	6,537	1,292	46,951
**Preoperative Prescription**	1,610 (16.5%)	1,712 (16.5%)	1,770 (16.7%)	1,303 (15.5%)	991 (15.2%)	201 (15.6%)	7,587 (16.2%)
**Refill up to 30 day after surgery, n(%)**	1,513 (15.5%)	1,457 (14.0%)	1,351 (12.8%)	1,100 (13.0%)	901 (13.8%)	145 (11.2%)	6,467 (13.8%)
**MME, mean (95% CI)**	264 (248 - 280)	271 (250 - 291)	233 (218 - 248)	213 (197 - 229)	177 (162 - 191)	199 (161 - 238)	239 (231 - 246)
**Refill 31-60 days after surgery, n(%)**	198 (2.0%)	191 (1.8%)	157 (1.5%)	78 (0.9%)	54 (0.8%)	7 (0.5%)	685 (1.5%)
**MME, mean (95% CI)**	248 (202 - 295)	250 (210 - 291)	189 (163 - 216)	180 (147 - 213)	124 (95 - 153)	294 (-145 - 733)	218 (199 - 238)
**Patients with continuous enrollment 180 days after surgery, N**	8,720	9,116	9,303	7,496	5,747		40,382[1]
**Refill 91-180 days after surgery, n(%)**	306 (3.5%)	298 (3.3%)	294 (3.2%)	196 (2.6%)	122 (2.1%)		1,216 (3.0%)
**MME, mean (95% CI)**	184 (160 - 207)	168 (150 - 186)	151 (137 - 164)	126 (107 - 144)	110 (98 - 122)		155 (146 - 164)

1. Excludes patients who had >91 and <180 days continuous enrollment after surgery in 2020; study period ended in May 2020

© 2024 Sutherland TN et al. *JAMA Network Open.*

eTable 6. Adjusted and Unadjusted Odds Ratios for Likelihood of Prescription Refill at Three Intervals

		Refill up to 30 Days after Surgery			Refill 31-60 Days after Surgery			Refill 91-180 Days after Surgery[1]		
Variable	Category	Events (Percent)	Unadjusted OR (95% CI)	Adjusted OR (95% CI)	Events (Percent)	Unadjusted OR (95% CI)	Adjusted OR (95% CI)	Events (Percent)	Unadjusted OR (95% CI)	Adjusted OR (95% CI)
Opioid Timing	Opioid 14-8 days prior	542/1,260 (43.0%)	6.39 (5.69 - 7.17)	5.98 (5.27 - 6.78)	40/1,260 (3.2%)	2.41 (1.74 - 3.34)	1.88 (1.33 - 2.64)	43/1,089 (3.9%)	1.40 (1.03 - 1.91)	1.17 (0.85 - 1.61)
	Opioid within 7 days prior	1,685/6,090 (27.7%)	3.24 (3.03 - 3.45)	2.68 (2.50 - 2.87)	115/6,090 (1.9%)	1.42 (1.15 - 1.74)	1.24 (1.01 - 1.54)	205/5,360 (3.8%)	1.35 (1.16 - 1.58)	1.31 (1.12 - 1.53)
	Opioid within 7 days after	4,035/38,159 (10.6%)	Ref	Ref	512/38,159 (1.3%)	Ref	Ref	968/33,933 (2.9%)	Ref	Ref
Age in Years	11	289/3,239 (8.9%)	Ref	Ref	9/3,239 (0.3%)	Ref	Ref	24/2,919 (0.8%)	Ref	Ref
	12	319/2,958 (10.8%)	1.23 (1.04 - 1.46)	1.26 (1.06 - 1.49)	17/2,958 (0.6%)	2.07 (0.92 - 4.66)	1.72 (0.76 - 3.87)	29/2,643 (1.1%)	1.34 (0.78 - 2.30)	1.28 (0.74 - 2.21)
	13	369/3,243 (11.4%)	1.31 (1.11 - 1.54)	1.39 (1.18 - 1.65)	21/3,243 (0.6%)	2.34 (1.07 - 5.11)	1.70 (0.78 - 3.74)	37/2,881 (1.3%)	1.57 (0.94 - 2.63)	1.44 (0.86 - 2.42)
	14	448/3,763 (11.9%)	1.38 (1.18 - 1.61)	1.54 (1.31 - 1.82)	37/3,763 (1.0%)	3.56 (1.72 - 7.39)	2.46 (1.18 - 5.14)	67/3,346 (2.0%)	2.46 (1.54 - 3.94)	2.20 (1.37 - 3.53)
	15	579/4,470 (13.0%)	1.52 (1.31 - 1.76)	1.75 (1.50 - 2.05)	60/4,470 (1.3%)	4.88 (2.42 - 9.85)	3.35 (1.65 - 6.81)	113/3,971 (2.8%)	3.53 (2.27 - 5.50)	3.13 (2.00 - 4.89)
	16	657/5,244 (12.5%)	1.46 (1.26 - 1.69)	1.71 (1.46 - 1.99)	77/5,244 (1.5%)	5.35 (2.68 - 10.67)	3.74 (1.86 - 7.53)	170/4,654 (3.7%)	4.57 (2.98 - 7.03)	4.04 (2.61 - 6.24)
	17	873/6,028 (14.5%)	1.73 (1.50 - 1.99)	2.08 (1.79 - 2.42)	103/6,028 (1.7%)	6.24 (3.15 - 12.34)	4.44 (2.23 - 8.84)	203/5,336 (3.8%)	4.77 (3.12 - 7.30)	4.13 (2.68 - 6.35)
	18	891/5,856 (15.2%)	1.83 (1.59 - 2.11)	2.25 (1.93 - 2.60)	128/5,856 (2.2%)	8.02 (4.07 - 15.77)	5.74 (2.89 - 11.37)	206/5,205 (4.0%)	4.97 (3.25 - 7.60)	4.22 (2.75 - 6.49)
	19	931/5,378 (17.3%)	2.14 (1.86 - 2.46)	2.63 (2.27 - 3.05)	106/5,378 (2.0%)	7.21 (3.65 - 14.26)	5.07 (2.55 - 10.10)	187/4,770 (3.9%)	4.92 (3.21 - 7.55)	4.10 (2.66 - 6.31)
	20	906/5,330 (17.0%)	2.09 (1.82 - 2.41)	2.72 (2.34 - 3.16)	109/5,330 (2.0%)	7.49 (3.79 - 14.80)	5.06 (2.54 - 10.10)	180/4,657 (3.9%)	4.85 (3.16 - 7.44)	3.93 (2.55 - 6.08)
Sex	Female	3,581/23,695 (15.1%)	1.27 (1.20 - 1.34)	1.06 (1.00 - 1.12)	374/23,695 (1.6%)	1.18 (1.01 - 1.37)	1.19 (1.01 - 1.40)	696/21,024 (3.3%)	1.24 (1.11 - 1.39)	1.16 (1.03 - 1.31)
Year	2015	1,474/9,497 (15.5%)	Ref	Ref	194/9,497 (2.0%)	Ref	Ref	306/8,720 (3.5%)	Ref	Ref
	2016	1,411/10,066 (14.0%)	0.89 (0.82 - 0.96)	0.83 (0.77 - 0.91)	189/10,066 (1.9%)	0.92 (0.75 - 1.12)	0.90 (0.73 - 1.11)	298/9,116 (3.3%)	0.93 (0.79 - 1.09)	0.89 (0.75 - 1.05)
	2017	1,318/10,255 (12.9%)	0.80 (0.74 - 0.87)	0.75 (0.69 - 0.82)	151/10,255 (1.5%)	0.72 (0.58 - 0.89)	0.71 (0.57 - 0.88)	294/9,303 (3.2%)	0.90 (0.76 - 1.06)	0.86 (0.73 - 1.02)
	2018	1,063/8,231 (12.9%)	0.81 (0.74 - 0.88)	0.77 (0.70 - 0.84)	76/8,231 (0.9%)	0.45 (0.34 - 0.58)	0.45 (0.34 - 0.59)	196/7,496 (2.6%)	0.74 (0.62 - 0.89)	0.72 (0.60 - 0.86)
	2019	875/6,348 (13.8%)	0.87 (0.79 - 0.95)	0.85 (0.77 - 0.93)	50/6,348 (0.8%)	0.38 (0.28 - 0.52)	0.38 (0.28 - 0.52)	122/5,747 (2.1%)	0.60 (0.48 - 0.74)	0.58 (0.47 - 0.72)
	2020	121/1,112 (10.9%)	0.66 (0.55 - 0.81)	0.63 (0.51 - 0.77)	7/1,112 (0.6%)	0.30 (0.14 - 0.65)	0.27 (0.13 - 0.58)			
Surgery	Incision and drainage	405/3,327 (12.2%)	0.94 (0.83 - 1.06)	0.81 (0.72 - 0.92)	112/3,327 (3.4%)	1.89 (1.47 - 2.43)	1.69 (1.31 - 2.17)	148/2,894 (5.1%)	1.61 (1.31 - 1.99)	1.56 (1.26 - 1.94)
	Dental surgery	1,300/7,910 (16.4%)	1.33 (1.22 - 1.46)	1.49 (1.36 - 1.64)	55/7,910 (0.7%)	0.38 (0.28 - 0.52)	0.43 (0.31 - 0.59)	171/7,177 (2.4%)	0.73 (0.60 - 0.89)	0.85 (0.69 - 1.04)
	Knee arthroscopy	1,036/8,068 (12.8%)	Ref	Ref	146/8,068 (1.8%)	Ref	Ref	232/7,173 (3.2%)	Ref	Ref

© 2024 Sutherland TN et al. *JAMA Network Open.*

	**Tonsillectomy**	1,321/5,825 (22.7%)	1.99 (1.82 - 2.18)	2.44 (2.22 - 2.69)	44/5,825 (0.8%)	0.41 (0.29 - 0.58)	0.51 (0.36 - 0.72)	119/5,225 (2.3%)	0.70 (0.56 - 0.87)	0.89 (0.71 - 1.12)
	**Appendectomy, laparoscopic or open**	256/6,227 (4.1%)	0.29 (0.25 - 0.34)	0.37 (0.32 - 0.42)	78/6,227 (1.3%)	0.69 (0.52 - 0.91)	0.78 (0.59 - 1.04)	153/5,564 (2.7%)	0.85 (0.69 - 1.04)	0.98 (0.80 - 1.21)
	**Hardware removal**	179/2,718 (6.6%)	0.48 (0.41 - 0.56)	0.48 (0.40 - 0.57)	32/2,718 (1.2%)	0.65 (0.44 - 0.95)	0.67 (0.45 - 1.01)	96/2,446 (3.9%)	1.22 (0.96 - 1.56)	1.22 (0.94 - 1.58)
	**Endoscopy**	10/77 (13.0%)	1.01 (0.52 - 1.98)	0.31 (0.16 - 0.62)	6/77 (7.8%)	4.59 (1.96 - 10.72)	2.43 (1.01 - 5.82)	6/63 (9.5%)	3.15 (1.34 - 7.38)	2.21 (0.93 - 5.24)
	**Cholecystectomy**	347/2,483 (14.0%)	1.10 (0.97 - 1.26)	0.91 (0.80 - 1.05)	42/2,483 (1.7%)	0.93 (0.66 - 1.32)	0.76 (0.53 - 1.09)	108/2,177 (5.0%)	1.56 (1.24 - 1.97)	1.38 (1.09 - 1.76)
	**Spinal fusion**	369/1,297 (28.5%)	2.70 (2.35 - 3.10)	3.08 (2.66 - 3.56)	50/1,297 (3.9%)	2.18 (1.57 - 3.02)	2.63 (1.87 - 3.71)	34/1,160 (2.9%)	0.90 (0.63 - 1.30)	1.04 (0.72 - 1.51)
	**Craniofacial surgery**	94/504 (18.7%)	1.56 (1.23 - 1.97)	1.12 (0.88 - 1.43)	6/504 (1.2%)	0.65 (0.29 - 1.49)	0.57 (0.25 - 1.29)	6/455 (1.3%)	0.40 (0.18 - 0.90)	0.35 (0.16 - 0.80)
	**Tympanoplasty**	47/990 (4.7%)	0.34 (0.25 - 0.46)	0.41 (0.31 - 0.56)	2/990 (0.2%)	0.11 (0.03 - 0.44)	0.15 (0.04 - 0.59)	19/840 (2.3%)	0.69 (0.43 - 1.11)	0.92 (0.57 - 1.48)
	**Lower extremity fracture repair**	472/1,282 (36.8%)	3.96 (3.47 - 4.51)	3.10 (2.70 - 3.56)	34/1,282 (2.7%)	1.48 (1.01 - 2.16)	1.50 (1.02 - 2.20)	24/985 (2.4%)	0.75 (0.49 - 1.14)	0.79 (0.51 - 1.21)
	**Circumcision**	36/899 (4.0%)	0.28 (0.20 - 0.40)	0.40 (0.29 - 0.57)	7/899 (0.8%)	0.43 (0.20 - 0.91)	0.63 (0.29 - 1.36)	13/804 (1.6%)	0.49 (0.28 - 0.86)	0.70 (0.40 - 1.24)
	**Orchiopexy**	36/1,082 (3.3%)	0.23 (0.17 - 0.33)	0.38 (0.27 - 0.53)	5/1,082 (0.5%)	0.25 (0.10 - 0.62)	0.46 (0.19 - 1.14)	12/965 (1.2%)	0.38 (0.21 - 0.68)	0.65 (0.36 - 1.18)
	**Craniotomy**	42/329 (12.8%)	0.99 (0.71 - 1.38)	1.13 (0.81 - 1.59)	12/329 (3.6%)	2.05 (1.13 - 3.74)	2.23 (1.22 - 4.09)	15/297 (5.1%)	1.59 (0.93 - 2.72)	1.72 (1.00 - 2.95)
	**Rhinoplasty**	67/848 (7.9%)	0.58 (0.45 - 0.75)	0.39 (0.30 - 0.51)	9/848 (1.1%)	0.58 (0.30 - 1.15)	0.50 (0.25 - 0.98)	24/755 (3.2%)	0.98 (0.64 - 1.51)	0.88 (0.57 - 1.36)
	**Breast surgery**	47/498 (9.4%)	0.71 (0.52 - 0.96)	0.33 (0.24 - 0.45)	6/498 (1.2%)	0.66 (0.29 - 1.50)	0.42 (0.18 - 0.97)	12/435 (2.8%)	0.85 (0.47 - 1.53)	0.55 (0.30 - 1.02)
	**Supracondylar fracture repair**	118/548 (21.5%)	1.86 (1.50 - 2.31)	2.14 (1.70 - 2.68)	4/548 (0.7%)	0.40 (0.15 - 1.08)	0.68 (0.25 - 1.85)	8/463 (1.7%)	0.53 (0.26 - 1.07)	0.89 (0.43 - 1.83)
	**Colectomy, partial or complete**	17/126 (13.5%)	1.06 (0.63 - 1.77)	1.13 (0.67 - 1.91)	7/126 (5.6%)	3.19 (1.46 - 6.96)	3.18 (1.44 - 7.01)	7/103 (6.8%)	2.18 (1.00 - 4.75)	2.24 (1.03 - 4.92)
	**Exploratory laparotomy**	17/96 (17.7%)	1.46 (0.86 - 2.48)	1.42 (0.83 - 2.45)	4/96 (4.2%)	2.36 (0.86 - 6.51)	2.10 (0.75 - 5.84)	3/82 (3.7%)	1.14 (0.36 - 3.63)	1.07 (0.34 - 3.44)
	**Bariatric surgery**	8/126 (6.3%)	0.46 (0.22 - 0.94)	0.26 (0.12 - 0.54)	1/126 (0.8%)	0.43 (0.06 - 3.13)	0.31 (0.04 - 2.24)	2/99 (2.0%)	0.62 (0.15 - 2.52)	0.51 (0.13 - 2.10)
	**Nuss bar insertion**	38/249 (15.3%)	1.22 (0.86 - 1.74)	1.38 (0.96 - 1.97)	5/249 (2.0%)	1.11 (0.45 - 2.74)	1.55 (0.62 - 3.84)	4/220 (1.8%)	0.55 (0.20 - 1.50)	0.71 (0.26 - 1.95)
**Comorbidities**	**Depression History**	114/671 (17.0%)	1.29 (1.05 - 1.58)	1.05 (0.84 - 1.31)	16/671 (2.4%)	1.66 (1.00 - 2.74)	1.22 (0.71 - 2.07)	29/589 (4.9%)	1.68 (1.15 - 2.46)	1.25 (0.84 - 1.86)
	**Anxiety History**	523/3,125 (16.7%)	1.28 (1.16 - 1.42)	1.20 (1.08 - 1.34)	63/3,125 (2.0%)	1.42 (1.10 - 1.85)	1.40 (1.06 - 1.86)	109/2,736 (4.0%)	1.37 (1.12 - 1.67)	1.23 (0.99 - 1.52)
	**Chronic pain diagnosis**	672/4,360 (15.4%)	1.16 (1.06 - 1.26)	1.32 (1.19 - 1.46)	85/4,360 (1.9%)	1.39 (1.10 - 1.74)	1.28 (0.99 - 1.65)	171/3,878 (4.4%)	1.57 (1.33 - 1.85)	1.40 (1.16 - 1.69)
	**History of Substance use disorder**	31/134 (23.1%)	1.89 (1.26 - 2.83)	1.62 (1.04 - 2.51)	5/134 (3.7%)	2.62 (1.07 - 6.42)	1.33 (0.53 - 3.37)	8/114 (7.0%)	2.44 (1.19 - 5.02)	1.57 (0.75 - 3.28)

© 2024 Sutherland TN et al. *JAMA Network Open.*

	**Overdose**	7/26 (26.9%)	2.31 (0.97 - 5.50)	2.64 (1.05 - 6.65)	1/26 (3.8%)	2.69 (0.36 - 19.89)	1.70 (0.21 - 13.56)	1/25 (4.0%)	1.34 (0.18 - 9.93)	0.91 (0.12 - 7.02)

1.. Refills up to 60 days includes 46,951 primary cohort patients and refills 91-180 days includes the subset of 40,382 patients who had enrollment through this period. Unable to include 2020 values as study period ended in May 2020.

© 2024 Sutherland TN et al. *JAMA Network Open.*

Appendix Table 7. Adjusted and Unadjusted Odds Ratios for Likelihood of Prescription Refill at Three Intervals, Sensitivity Analysis with MME Ranges

		Refill up to 30 Days after Surgery			Refill 31-60 Days after Surgery			Refill 91-180 Days after Surgery[1]		
**Variable**	**Category**	**Events (Percent)**	**Unadjusted OR (95% CI)**	**Adjusted OR (95% CI)**	**Events (Percent)**	**Unadjusted OR (95% CI)**	**Adjusted OR (95% CI)**	**Events (Percent)**	**Unadjusted OR (95% CI)**	**Adjusted OR (95% CI)**
**Opioid Timing**	**Opioid 14-8 days prior**	542/1,260 (43.0%)	6.39 (5.69 - 7.17)	5.63 (4.96 - 6.39)	40/1,260 (3.2%)	2.41 (1.74 - 3.34)	1.96 (1.39 - 2.77)	43/1,089 (3.9%)	1.40 (1.03 - 1.91)	1.19 (0.86 - 1.63)
	**Opioid within 7 days prior**	1,685/6,090 (27.7%)	3.24 (3.03 - 3.45)	2.63 (2.45 - 2.81)	115/6,090 (1.9%)	1.42 (1.15 - 1.74)	1.27 (1.02 - 1.56)	205/5,360 (3.8%)	1.35 (1.16 - 1.58)	1.32 (1.12 - 1.54)
	**Opioid within 7 days after**	4,035/38,159 (10.6%)	Ref	Ref	512/38,159 (1.3%)	Ref	Ref	968/33,933 (2.9%)	Ref	Ref
**MME Dispensed**	**<25**	43/1,089 (3.9%)	1.40 (1.03 - 1.91)	1.19 (0.86 - 1.63)	3/449 (0.7%)	0.57 (0.18 - 1.81)	0.75 (0.23 - 2.41)	9/376 (2.4%)	0.85 (0.43 - 1.68)	1.10 (0.55 - 2.21)
	**26-60**	205/5,360 (3.8%)	1.35 (1.16 - 1.58)	1.32 (1.12 - 1.54)	56/4,759 (1.2%)	Ref	Ref	115/4,087 (2.8%)	Ref	Ref
	**61-100**	968/33,933 (2.9%)	Ref	Ref	113/8,436 (1.3%)	1.14 (0.83 - 1.57)	1.09 (0.78 - 1.51)	213/7,339 (2.9%)	1.03 (0.82 - 1.30)	0.96 (0.76 - 1.21)
	**101-200**	2,031/14,104 (14.4%)	1.20 (1.08 - 1.32)	0.88 (0.79 - 0.98)	179/14,104 (1.3%)	1.08 (0.80 - 1.46)	1.09 (0.79 - 1.50)	367/12,521 (2.9%)	1.04 (0.84 - 1.29)	0.97 (0.77 - 1.21)
	**>200**	2,548/17,761 (14.3%)	1.19 (1.08 - 1.31)	0.61 (0.55 - 0.69)	316/17,761 (1.8%)	1.52 (1.14 - 2.02)	1.41 (1.01 - 1.95)	512/16,059 (3.2%)	1.14 (0.93 - 1.40)	1.07 (0.85 - 1.36)
**Age in Years**	**11**	289/3,239 (8.9%)	Ref	Ref	9/3,239 (0.3%)	Ref	Ref	24/2,919 (0.8%)	Ref	Ref
	**12**	319/2,958 (10.8%)	1.23 (1.04 - 1.46)	1.27 (1.06 - 1.51)	17/2,958 (0.6%)	2.07 (0.92 - 4.66)	1.70 (0.75 - 3.83)	29/2,643 (1.1%)	1.34 (0.78 - 2.30)	1.29 (0.75 - 2.22)
	**13**	369/3,243 (11.4%)	1.31 (1.11 - 1.54)	1.42 (1.20 - 1.68)	21/3,243 (0.6%)	2.34 (1.07 - 5.11)	1.67 (0.76 - 3.67)	37/2,881 (1.3%)	1.57 (0.94 - 2.63)	1.45 (0.86 - 2.43)
	**14**	448/3,763 (11.9%)	1.38 (1.18 - 1.61)	1.58 (1.34 - 1.86)	37/3,763 (1.0%)	3.56 (1.72 - 7.39)	2.41 (1.15 - 5.02)	67/3,346 (2.0%)	2.46 (1.54 - 3.94)	2.21 (1.38 - 3.54)
	**15**	579/4,470 (13.0%)	1.52 (1.31 - 1.76)	1.81 (1.54 - 2.11)	60/4,470 (1.3%)	4.88 (2.42 - 9.85)	3.24 (1.59 - 6.59)	113/3,971 (2.8%)	3.53 (2.27 - 5.50)	3.13 (2.00 - 4.90)
	**16**	657/5,244 (12.5%)	1.46 (1.26 - 1.69)	1.76 (1.51 - 2.05)	77/5,244 (1.5%)	5.35 (2.68 - 10.67)	3.61 (1.79 - 7.27)	170/4,654 (3.7%)	4.57 (2.98 - 7.03)	4.04 (2.61 - 6.24)
	**17**	873/6,028 (14.5%)	1.73 (1.50 - 1.99)	2.15 (1.85 - 2.49)	103/6,028 (1.7%)	6.24 (3.15 - 12.34)	4.27 (2.14 - 8.51)	203/5,336 (3.8%)	4.77 (3.12 - 7.30)	4.13 (2.68 - 6.35)
	**18**	891/5,856 (15.2%)	1.83 (1.59 - 2.11)	2.34 (2.01 - 2.71)	128/5,856 (2.2%)	8.02 (4.07 - 15.77)	5.47 (2.76 - 10.86)	206/5,205 (4.0%)	4.97 (3.25 - 7.60)	4.21 (2.74 - 6.48)
	**19**	931/5,378 (17.3%)	2.14 (1.86 - 2.46)	2.77 (2.38 - 3.21)	106/5,378 (2.0%)	7.21 (3.65 - 14.26)	4.80 (2.41 - 9.58)	187/4,770 (3.9%)	4.92 (3.21 - 7.55)	4.07 (2.64 - 6.29)
	**20**	906/5,330 (17.0%)	2.09 (1.82 - 2.41)	2.89 (2.49 - 3.36)	109/5,330 (2.0%)	7.49 (3.79 - 14.80)	4.78 (2.39 - 9.55)	180/4,657 (3.9%)	4.85 (3.16 - 7.44)	3.91 (2.53 - 6.05)
**Sex**	**Female**	3,581/23,695 (15.1%)	1.27 (1.20 - 1.34)	1.06 (1.00 - 1.13)	374/23,695 (1.6%)	1.18 (1.01 - 1.37)	1.20 (1.02 - 1.41)	696/21,024 (3.3%)	1.24 (1.11 - 1.39)	1.16 (1.03 - 1.31)
**Year**	**2015**	1,474/9,497 (15.5%)	Ref	Ref	194/9,497 (2.0%)	Ref	Ref	306/8,720 (3.5%)	Ref	Ref
	**2016**	1,411/10,066 (14.0%)	0.89 (0.82 - 0.96)	0.83 (0.76 - 0.90)	189/10,066 (1.9%)	0.92 (0.75 - 1.12)	0.91 (0.74 - 1.11)	298/9,116 (3.3%)	0.93 (0.79 - 1.09)	0.89 (0.76 - 1.05)
	**2017**	1,318/10,255 (12.9%)	0.80 (0.74 - 0.87)	0.73 (0.67 - 0.80)	151/10,255 (1.5%)	0.72 (0.58 - 0.89)	0.72 (0.58 - 0.90)	294/9,303 (3.2%)	0.90 (0.76 - 1.06)	0.87 (0.73 - 1.02)
	**2018**	1,063/8,231 (12.9%)	0.81 (0.74 - 0.88)	0.71 (0.65 - 0.78)	76/8,231 (0.9%)	0.45 (0.34 - 0.58)	0.47 (0.36 - 0.62)	196/7,496 (2.6%)	0.74 (0.62 - 0.89)	0.73 (0.60 - 0.88)
	**2019**	875/6,348 (13.8%)	0.87 (0.79 - 0.95)	0.72 (0.65 - 0.80)	50/6,348 (0.8%)	0.38 (0.28 - 0.52)	0.43 (0.31 - 0.60)	122/5,747 (2.1%)	0.60 (0.48 - 0.74)	0.60 (0.48 - 0.75)
	**2020**	121/1,112 (10.9%)	0.66 (0.55 - 0.81)	0.53 (0.43 - 0.66)	7/1,112 (0.6%)	0.30 (0.14 - 0.65)	0.30 (0.14 - 0.66)			
**Surgery**	**Incision and drainage**	405/3,327 (12.2%)	0.94 (0.83 - 1.06)	0.67 (0.59 - 0.77)	112/3,327 (3.4%)	1.89 (1.47 - 2.43)	1.98 (1.49 - 2.63)	148/2,894 (5.1%)	1.61 (1.31 - 1.99)	1.63 (1.29 - 2.07)

© 2024 Sutherland TN et al. *JAMA Network Open.*

	**Dental surgery**	1,300/7,910 (16.4%)	1.33 (1.22 - 1.46)	1.45 (1.32 - 1.59)	55/7,910 (0.7%)	0.38 (0.28 - 0.52)	0.44 (0.32 - 0.61)	171/7,177 (2.4%)	0.73 (0.60 - 0.89)	0.86 (0.70 - 1.05)
	**Knee arthroscopy**	1,036/8,068 (12.8%)	Ref	Ref	146/8,068 (1.8%)	Ref	Ref	232/7,173 (3.2%)	Ref	Ref
	**Tonsillectomy**	1,321/5,825 (22.7%)	1.99 (1.82 - 2.18)	2.59 (2.35 - 2.85)	44/5,825 (0.8%)	0.41 (0.29 - 0.58)	0.49 (0.35 - 0.69)	119/5,225 (2.3%)	0.70 (0.56 - 0.87)	0.88 (0.70 - 1.10)
	**Appendectomy, laparoscopic or open**	256/6,227 (4.1%)	0.29 (0.25 - 0.34)	0.32 (0.27 - 0.37)	78/6,227 (1.3%)	0.69 (0.52 - 0.91)	0.87 (0.65 - 1.17)	153/5,564 (2.7%)	0.85 (0.69 - 1.04)	1.02 (0.82 - 1.27)
	**Hardware removal**	179/2,718 (6.6%)	0.48 (0.41 - 0.56)	0.44 (0.37 - 0.52)	32/2,718 (1.2%)	0.65 (0.44 - 0.95)	0.72 (0.48 - 1.08)	96/2,446 (3.9%)	1.22 (0.96 - 1.56)	1.24 (0.96 - 1.62)
	**Endoscopy**	10/77 (13.0%)	1.01 (0.52 - 1.98)	0.27 (0.13 - 0.54)	6/77 (7.8%)	4.59 (1.96 - 10.72)	2.74 (1.14 - 6.61)	6/63 (9.5%)	3.15 (1.34 - 7.38)	2.30 (0.96 - 5.47)
	**Cholecystectomy**	347/2,483 (14.0%)	1.10 (0.97 - 1.26)	0.81 (0.70 - 0.93)	42/2,483 (1.7%)	0.93 (0.66 - 1.32)	0.84 (0.58 - 1.20)	108/2,177 (5.0%)	1.56 (1.24 - 1.97)	1.43 (1.12 - 1.83)
	**Spinal fusion**	369/1,297 (28.5%)	2.70 (2.35 - 3.10)	3.39 (2.92 - 3.92)	50/1,297 (3.9%)	2.18 (1.57 - 3.02)	2.47 (1.75 - 3.49)	34/1,160 (2.9%)	0.90 (0.63 - 1.30)	1.02 (0.70 - 1.48)
	**Craniofacial surgery**	94/504 (18.7%)	1.56 (1.23 - 1.97)	1.02 (0.80 - 1.31)	6/504 (1.2%)	0.65 (0.29 - 1.49)	0.61 (0.27 - 1.40)	6/455 (1.3%)	0.40 (0.18 - 0.90)	0.36 (0.16 - 0.82)
	**Tympanoplasty**	47/990 (4.7%)	0.34 (0.25 - 0.46)	0.36 (0.27 - 0.49)	2/990 (0.2%)	0.11 (0.03 - 0.44)	0.16 (0.04 - 0.66)	19/840 (2.3%)	0.69 (0.43 - 1.11)	0.96 (0.59 - 1.55)
	**Lower extremity fracture repair**	472/1,282 (36.8%)	3.96 (3.47 - 4.51)	2.98 (2.59 - 3.43)	34/1,282 (2.7%)	1.48 (1.01 - 2.16)	1.55 (1.05 - 2.28)	24/985 (2.4%)	0.75 (0.49 - 1.14)	0.80 (0.52 - 1.23)
	**Circumcision**	36/899 (4.0%)	0.28 (0.20 - 0.40)	0.34 (0.24 - 0.48)	7/899 (0.8%)	0.43 (0.20 - 0.91)	0.73 (0.33 - 1.58)	13/804 (1.6%)	0.49 (0.28 - 0.86)	0.73 (0.41 - 1.30)
	**Orchiopexy**	36/1,082 (3.3%)	0.23 (0.17 - 0.33)	0.32 (0.23 - 0.45)	5/1,082 (0.5%)	0.25 (0.10 - 0.62)	0.53 (0.21 - 1.32)	12/965 (1.2%)	0.38 (0.21 - 0.68)	0.68 (0.38 - 1.24)
	**Craniotomy**	42/329 (12.8%)	0.99 (0.71 - 1.38)	1.09 (0.78 - 1.53)	12/329 (3.6%)	2.05 (1.13 - 3.74)	2.28 (1.24 - 4.18)	15/297 (5.1%)	1.59 (0.93 - 2.72)	1.72 (1.00 - 2.96)
	**Rhinoplasty**	67/848 (7.9%)	0.58 (0.45 - 0.75)	0.36 (0.28 - 0.47)	9/848 (1.1%)	0.58 (0.30 - 1.15)	0.53 (0.27 - 1.05)	24/755 (3.2%)	0.98 (0.64 - 1.51)	0.91 (0.59 - 1.39)
	**Breast surgery**	47/498 (9.4%)	0.71 (0.52 - 0.96)	0.32 (0.23 - 0.45)	6/498 (1.2%)	0.66 (0.29 - 1.50)	0.42 (0.18 - 0.98)	12/435 (2.8%)	0.85 (0.47 - 1.53)	0.56 (0.30 - 1.02)
	**Supracondylar fracture repair**	118/548 (21.5%)	1.86 (1.50 - 2.31)	1.92 (1.52 - 2.41)	4/548 (0.7%)	0.40 (0.15 - 1.08)	0.73 (0.27 - 1.99)	8/463 (1.7%)	0.53 (0.26 - 1.07)	0.91 (0.44 - 1.87)
	**Colectomy, partial or complete**	17/126 (13.5%)	1.06 (0.63 - 1.77)	1.04 (0.62 - 1.76)	7/126 (5.6%)	3.19 (1.46 - 6.96)	3.37 (1.53 - 7.44)	7/103 (6.8%)	2.18 (1.00 - 4.75)	2.29 (1.04 - 5.02)
	**Exploratory laparotomy**	17/96 (17.7%)	1.46 (0.86 - 2.48)	1.32 (0.77 - 2.28)	4/96 (4.2%)	2.36 (0.86 - 6.51)	2.23 (0.80 - 6.20)	3/82 (3.7%)	1.14 (0.36 - 3.63)	1.09 (0.34 - 3.51)
	**Bariatric surgery**	8/126 (6.3%)	0.46 (0.22 - 0.94)	0.24 (0.11 - 0.50)	1/126 (0.8%)	0.43 (0.06 - 3.13)	0.33 (0.05 - 2.39)	2/99 (2.0%)	0.62 (0.15 - 2.52)	0.53 (0.13 - 2.15)
	**Nuss bar insertion**	38/249 (15.3%)	1.22 (0.86 - 1.74)	1.47 (1.02 - 2.10)	5/249 (2.0%)	1.11 (0.45 - 2.74)	1.48 (0.60 - 3.68)	4/220 (1.8%)	0.55 (0.20 - 1.50)	0.70 (0.26 - 1.92)
**Comorbidities**	**Depression History**	114/671 (17.0%)	1.29 (1.05 - 1.58)	1.05 (0.84 - 1.32)	16/671 (2.4%)	1.66 (1.00 - 2.74)	1.22 (0.71 - 2.07)	29/589 (4.9%)	1.68 (1.15 - 2.46)	1.25 (0.84 - 1.86)
	**Anxiety History**	523/3,125 (16.7%)	1.28 (1.16 - 1.42)	1.20 (1.08 - 1.34)	63/3,125 (2.0%)	1.42 (1.10 - 1.85)	1.40 (1.06 - 1.86)	109/2,736 (4.0%)	1.37 (1.12 - 1.67)	1.23 (0.99 - 1.52)
	**Chronic pain diagnosis**	672/4,360 (15.4%)	1.16 (1.06 - 1.26)	1.31 (1.19 - 1.45)	85/4,360 (1.9%)	1.39 (1.10 - 1.74)	1.27 (0.98 - 1.65)	171/3,878 (4.4%)	1.57 (1.33 - 1.85)	1.40 (1.16 - 1.69)

© 2024 Sutherland TN et al. *JAMA Network Open.*

	**History of Substance use disorder**	31/134 (23.1%)	1.89 (1.26 - 2.83)	1.59 (1.02 - 2.46)	5/134 (3.7%)	2.62 (1.07 - 6.42)	1.35 (0.53 - 3.41)	8/114 (7.0%)	2.44 (1.19 - 5.02)	1.59 (0.76 - 3.32)
	**Overdose**	7/26 (26.9%)	2.31 (0.97 - 5.50)	2.58 (1.02 - 6.51)	1/26 (3.8%)	2.69 (0.36 - 19.89)	1.75 (0.22 - 13.89)	1/25 (4.0%)	1.34 (0.18 - 9.93)	0.91 (0.12 - 7.05)

1.. Refills up to 60 days includes 47,369 primary cohort patients and refills 91-180 days includes the subset of 40,382 patients who had enrollment through this period. Unable to include 2020 values as study period ended in May 2020.

© 2024 Sutherland TN et al. *JAMA Network Open.*
