# Supplementary material for: Preoperative vs Postoperative Opioid Prescriptions and Prolonged Opioid Refills Among US Youths
Source: JAMA Netw Open. 2024 Jul 5;7(7):e2420370. doi: 10.1001/jamanetworkopen.2024.20370 (PMC11227082; doi:10.1001/jamanetworkopen.2024.20370)
Supplement: Supplement 2. — Data Sharing Statement [file jamanetwopen-e2420370-s002.pdf]

Data Sharing Statement

Sutherland. Preoperative vs Postoperative Opioid Prescriptions and Prolonged Opioid Refills Among US Youths. *JAMA Netw Open*. Published July 05, 2024.
doi:10.1001/jamanetworkopen.2024.20370

Data
Data available: No

Additional Information
Explanation for why data not available: The Optum-University of Pennsylvania agreement does not permit data sharing; however, we are able to share our codebook for reasonable requests.
